# 5-Year Mortality After Complex Displaced Proximal Humerus Fractures in Elderly Patients: Conservative Versus Reverse Total Shoulder Arthroplasty

**DOI:** 10.3390/jcm14010167

**Published:** 2024-12-31

**Authors:** Gal Maman, Ofir Chechik, Efi Kazum, Assaf Bivas, Eran Maman, Dani Rotman

**Affiliations:** 1 Tel-Aviv Sourasky Medical Center, Faculty of Medicine, Tel-Aviv University, Tel Aviv 6423906, Israeldrotman@laniado.org.il (D.R.); 2 Department of Biomedical Sciences, Humanitas University, 20072 Milan, Italy; 3 Laniado Hospital, Adelson Faculty of Medicine, Ariel Univesity, Netanya 4244916, Israel

**Keywords:** reverse total shoulder arthroplasty, proximal humerus fracture, elderly patients, conservative treatment, surgical treatment, 5-year follow-up, mortality rate

## Abstract

**Background:** The mortality rate following proximal humerus fractures (PHFs) in elderly patients is increased, but currently, there are no medium-term studies comparing mortality following treatment with Reverse Total Shoulder Arthroplasty (RTSA) to non-surgical treatment. **Methods:** This retrospective study compares two groups of elderly patients (aged 75 to 95 at the time of injury) who were diagnosed with PHFs. A total of 79 patients (mean age: 83.1 ± 4.6) were treated conservatively between 2008 and 2010, a time when RTSA was not yet considered a treatment option, and 81 patients (mean age: 82.4 ± 4.4) underwent RTSA between 2012 and 2017. Also, 1-month, 1-year, and 5-year mortality rates were recorded. **Results:** The 1-month, 1-year, and 5-year mortality rates were 1.2%, 7.4%, and 33.3% in the RTSA group and 2.5%, 11.4%, and 38.0% in the non-surgical treatment group (*p* = 0.98, *p* = 0.55, *p* = 0.65). A subgroup analysis revealed that the mild difference between groups can be attributed to male patients only. **Conclusions:** This study explored the impact of RTSA versus non-surgical treatment on mortality in elderly patients with PHFs and found similar mortality rates over five years. Better segmentation of the patient population may reveal subgroups with different mortality patterns.

## 1. Introduction

PHFs are the third most common fragility fracture in elderly patients [1]. They are twice as common in females than males [2], and they occur more frequently with increasing age [3]. PHFs may cause impairment of quality of life and are associated with increased mortality rates in elderly patients compared to the general healthy population [4,5].

PHFs can be treated non-surgically or surgically, depending on the fracture type, patient health, and functional status. Nonsurgical treatment, immobilization followed by physiotherapy and pain management, is usually preferred [6]. Surgical treatment includes open reduction and internal fixation (ORIF), Closed Reduction and internal or external fixation, hemiarthroplasty (HA), and reverse total shoulder arthroplasty (RTSA).

Comparisons in mortality rates were made between surgical and non-surgical treatments with varying results. In a retrospective review of 319 elderly patients with PHFs, Myeroff et al. found a lower mortality rate in patients who underwent surgical treatment. Still, they attributed this difference to their better pre-injury health [7]. Similar results were recorded by Klute et al. in a retrospective review of 522 cases with a minimum follow-up of 5 years [8]. Other studies showed no difference, such as Rabi et al., which performed a systematic review and meta-analysis of six randomized controlled trials comparing operative vs. non-operative treatment of PHFs in elderly patients [9].

Our previous study [10] examined whether treatment with RTSA decreases 1-year mortality rate among elderly patients with PHF, compared to non-surgical treatment, revealed a trend of reduced mortality rate among RTSA-treated patients, although not statistically significant. We hypothesized that a more definitive result would be seen with a longer follow-up of 5 years.

## 2. Materials and Methods

After institutional review board approval, a retrospective observational comparative study was performed. The RTSA group included all patients over 75 diagnosed with PHF and treated with RTSA in one medical center between 2012 and 2017. During this period, RTSA was performed for most surgically treated PHF in this age group. Other treatment options, namely CRIF or ORIF, were offered only to the rare patients with displaced simple (2-part) fractures and good bone quality. Exclusion criteria from this group were concomitant lower limb fracture and medically unstable patients before surgery (American Society of Anesthesiologists [ASA] score > 3).

All RTSA surgeries were performed by one of six fellowship-trained shoulder surgeons, usually within a week of the fracture. Several implant systems were used during the study period, but they were all Gramont-style RTSA implants. Patients were usually discharged the day after the surgery and were instructed to use a sling for six weeks.

The control group consisted of patients over 75 years of age with displaced PHFs (Neer 4, 3, or 2 with complete displacement of the humeral head) between 2008 and 2010. At this period, RTSA was not yet considerable as a treatment option for PHFs in our institution, and almost all elderly patients were treated conservatively. To include only patients who would have been treated with RTSA by current practices, exclusion criteria from the control group included (1) unstable medical condition at presentation; (2) dependent, inactive, and/or cognitively impaired patients; (3) patients with insufficient available data to determine their medical status retrospectively; (4) age over 95 years. Other exclusion criteria were patients who had received surgical treatment or patients with concomitant lower limb fractures.

The mortality rates examined were for 1 month, 1 year, and 5 years. Mortality was determined using the Israeli Ministry of the Interior database.

### Statistical Analysis

Categorical variables were compared using the Fisher exact test or the χ2 test. Quantitative variables were summarized by mean and standard error of the mean. Numerical variables (The Charlson comorbidity index) were clustered into groups to facilitate analysis and improve interpretability. Parametric variable means were compared using Student’s *t*-test, and in the case of nonparametric variables, the groups were compared using the Mann–Whitney test. Moreover, 95% confidence intervals were calculated when sample proportions followed a normal distribution (one and 5-year mortality rates).

Kaplan–Meier curves were constructed to estimate the distribution of survival, and comparison between the groups was assessed by a log-rank test. A *p*-value of <0.05 was considered statistically significant for all tests. Statistical analysis was performed using R statistical software version 4.1.0.

## 3. Results

A total of 87 patients met the inclusion criteria for the surgical treatment group, of which 6 were excluded (2 had a concomitant tibial fracture, 4 were classified as ASA 4, and 1 was a tourist lost to follow-up), leaving 81 patients. A total of 182 patients met the inclusion criteria for the non-surgical treatment group, of which 103 were excluded (44 lacked sufficient medical data; 29 were dependent, inactive, and/or cognitively impaired; 13 had surgical treatment using one of the following techniques: external fixation, ORIF, or Hemiarthroplasty; 8 had a concomitant lower limb fracture, 5 were medically unstable at presentation, and 4 were aged above 95), leaving 79 patients.

In summary, 79 patients were treated conservatively, and 81 patients were treated with RTSA; 80.6% were females.

The patients’ demographics and medical backgrounds are summarized in Table 1. Differences between the two groups were minor; therefore, we did not use propensity score matching for the statistical analysis.

There were no significant differences between groups in one-month, one-year, or five-year mortality. Subgroup analysis of male patients also found no significant differences in mortality rates between groups. Mortality rates of the entire cohort, both groups and male patients only, with 95% confidence intervals for the one and 5-years results, are described in Table 2.

## 4. Discussion

This study examined whether RTSA can reduce mortality in elderly patients suffering from complex PHFs compared to non-surgical treatment over a 5-year follow-up period. The results showed similar mortality rates in both RTSA and non-surgical treatment groups and did not support our hypothesis of reduced mortality rates following RTSA.

This study also examined whether surgical treatment could mitigate mortality rates in male patients, given the known association of male sex with elevated mortality and heightened complications following surgical treatment for proximal humeral fractures [11,12]. Our prior research also prompted this subgroup analysis, revealing a large, albeit statistically insignificant, trend of reduced mortality among males undergoing RTSA. The current study, with a somewhat larger population and a longer 5-year follow-up, shows similar results: a trend towards reduced mortality in the male population treated with RTSA, albeit not statistically significant.

Recent studies showed different results and conclusions regarding the optimal treatment for PHFs in elderly patients. Some studies show no significant difference in the survival rate between nonoperative and operative treatment. A study conducted by Rabi et al. performed a systematic literature search of six randomized controlled trials, comparing operative and non-operative treatment to PHFs in 287 elderly patients, demonstrating no statistically significant difference in mortality outcome [9]. A similar conclusion was shown by Handoll et al., which assessed from 8 studies, including 646 participants, the effects of treatment and rehabilitation interventions for proximal humeral fractures [13]. The PROFHER trial, a large study conducted to evaluate the clinical effectiveness of surgical compared with non-surgical treatment of PHFs in elderly patients, with a 2-year follow-up and 250 participants, also aligned with our conclusions regarding the mortality rate [14]. Even though the mortality rate was not the main focus of these studies, they supported the previously mentioned conclusion. Nonetheless, these studies examined all kinds of surgical treatments and were not focused on RTSA, which has a lower complication rate and better results than other surgical interventions in this population, namely ORIF [15] and Hemiarthroplasty [16].

On the contrary, some studies show a lower mortality rate with surgical treatment (not specifically RTSA) for PHFs. For example, a nationwide study conducted in Sweden of 147,692 patients with a follow-up of up to 17 years [17] showed that the mortality rate was lower in the surgically treated group. Although this study presented a focused comparison of mortality rate between surgical treatment and conservative treatment in patients aged 80+, it showed data concerning all types of surgical treatments, and the information on patients’ comorbidities before surgical or non-surgical treatment was not discussed. A similar conclusion was shown by Duey et al., who analyzed treatment outcomes between operative and non-operative in 49,072 patients with PHFs with a 1-year follow-up [18]. However, a significant limitation is that male sex, older age, increased CCI, and high Hospital Frailty Risk Score (HFRS) were associated with decreased odds of operative treatment. While both studies associate decreased mortality with surgical compared to non-surgical treatment, they have similar limitations and should probably be interpreted with reverse causality—the healthier patients are treated surgically, and that is the reason for their better longevity.

Lastly, some studies present a higher mortality rate with surgical treatment; Garcia-Reza A. et al. collected data from 638 patients with PHFs and focused mainly on the effect of high CCI on the mortality rate for the type of treatment. A comparison was made between patients with CCI higher than five treated surgically and non-surgically and showed higher mortality in the former [19]. A similar study by Fernández-Cortiñas AB et al. that also focused on patients with high CCI, presented similar conclusions regarding treatment clinical outcomes and the effect CCI > 5 has on mortality rate among surgically and non-surgically treated patients [20]. However, both studies, when focusing on all patients and not only those with CCI > 5, showed no significant difference in mortality rate between surgical and non-surgical treatment.

These supposedly conflicting results can be summarized coherently: proximal humerus fracture in elderly patients, like other fragility fractures, increases 1- and 5-year mortality in the general population [5]. However, this increased mortality primarily happens in two specific subpopulations: male patients [21] and fragile patients. For female patients, which account for the vast majority of patients with PHF, there is no increased mortality to begin with, so surgical treatment does not change mortality. Unfortunately, surgically treating fragile patients does not reduce their mortality risk but increases it in the short term. Healthy male patients may benefit from prompt surgery with RTSA, but the small number of such patients in our study prevents drawing clear conclusions.

The rationale behind our subgroup analysis for male patients arises from the observations that males exhibit a lower prevalence of frailty but higher mortality rates than females with similar conditions [22,23]. This difference is particularly evident in osteoporosis and fragility fractures, which are more common in females but have a more severe impact on male mortality [24]. In the case of hip fractures, where osteoporosis and frailty are critical factors, prompt surgical treatment was proven to improve survival outcomes significantly [25]. These findings led us to the notion that surgical treatment with RTSA, which facilitates faster rehabilitation, might greatly benefit male patients, who, as previously noted, tend to experience worse outcomes than females.

The main strength of this study was the long follow-up period of 5 years. An additional strength was the almost equal number of non-surgical patients vs. surgically treated group of RTSA-only patients (79 and 81, respectively) and the fact that these patients shared very similar characteristics, giving a higher creditability to the results obtained. The weaknesses of this study included the following: (1) a low study population, which could limit the reliability of the results procured; (2) there was a temporal difference between the two groups: conservative patients from 2008 to 2010 and RTSA patients from 2012 to 2017, which could lead to a bias as medical care is changing and constantly improving; (3) the large number of excluded patients in the nonsurgical group could lead to a selection bias; (4) no quality-of-life measurements were available for the research population, which is mandatory to the decision whether to suggest a surgical or non-surgical treatment; (5) the specific clustering of the Charlson comorbidity index into groups of 2 points each was chosen arbitrarily.

## 5. Conclusions

With a 5-year follow-up, elderly individuals who underwent RTSA for PHFs had an insignificant difference in mortality rate compared to patients treated non-surgically. Therefore, changes in mortality risk should not be considered when patients and surgeons choose between conservative treatment and RTSA in these cases. Nonetheless, better segmentation of the patient population may reveal subgroups with different mortality patterns. Specifically, male patients may benefit from reduced mortality after surgery, whereas frail patients may suffer from increased mortality.

## Figures and Tables

**Figure 1:**
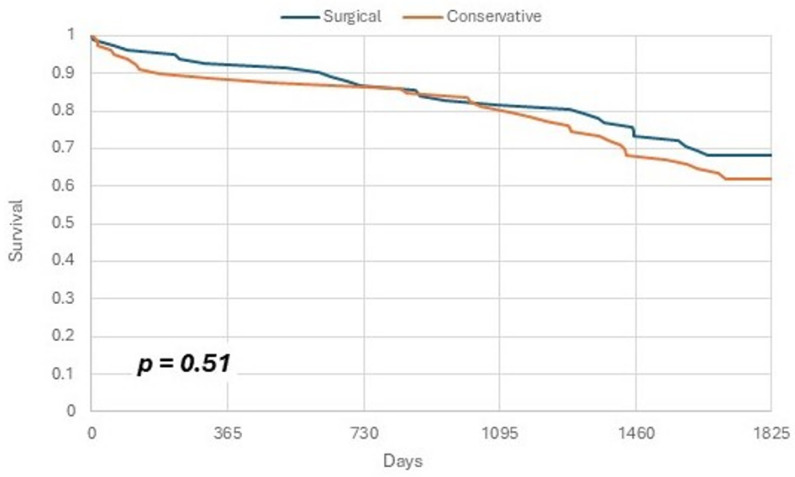
Survival analysis (Kaplan–Meier curves).

**Table 1:** Demographic characteristics and medical background of the two patient groups.

	Conservative Treatment (*n* = 79)	Surgical Treatment (RTSA; *n* = 81)	*p*-Value
Gender	F = 63 (80%)	F = 66 (81%)	0.94
Age	83.1 ± 4.6 M 84.2; F 82.8	82.4 ± 4.4 M 83.5; F 82.1	0.32
CCI			
3–4	30 (38%)	41 (51%)	
5–6	30 (38%)	31 (38%)	0.15
7–8	17 (21%)	8 (10%)	
9–10	2 (3%)	1 (1%)	
Mean	5.4	4.7	

CCI: Charlon comorbidity index.

**Table 2:** Mortality rates for patients with PHFs.

	1-Month Mortality	1-Year Mortality	5-Year Mortality
Entire cohort (*n* = 160)	3 (1.9%)	15 (9.4%) 95CI [4.9–13.9%]	57 (35.6%) 95CI [28.2–43.0%]
RTSA (*n* = 81)	1 (1.2%)	6 (7.4%) 95CI [1.7–13.1%]	27 (33.3%) 95CI [23.0–43.6%]
Conservative (*n* = 79)	2 (2.5%)	9 (11.4%) 95CI [4.4–18.4%]	30 (38.0%) 95CI [27.3–48.7%]
*p*-value	0.98	0.55	0.65
Male only RTSA (*n* = 15)	0	1 (8.3%)	6 (40.0%)
Male only Conservative (*n* = 18)	1 (5.6%)	3 (16.7%)	9 (50%)
*p*-value	1	0.73	0.82

Survival analysis (Kaplan–Meier curves) is depicted in Figure 1.

## Data Availability

The data presented in this study are available upon request from the corresponding author.
